# Toward trustworthy diagnostic AI: debiasing and grounding large language models for seizure semiology interpretation

**DOI:** 10.3389/fneur.2026.1990185

**Published:** 2026-10-29

**Authors:** Xiaolei Yuan, Xinyue Zhang, Hui Xu, Qing Ye, Canxing Yuan, Hui Li

**Affiliations:** 1 Unit 1, Department of Neurology, Longhua Hospital, Shanghai University of Traditional Chinese Medicine, Shanghai, China; 2 Shanghai Minhang District Traditional Chinese Medicine Hospital, Shanghai, China

**Keywords:** hallucination detection, large language models, retrieval-augmented generation, seizure semiology localization, self-consistency

## Abstract

Large language models (LLMs) have been proposed to map free-text seizure semiology to seizure-onset-zone (SOZ) likelihoods, but it remains unclear whether inference-time interventions can improve on the original SemioLLM benchmark without additional training. We evaluated two such interventions, candidate-order permutation within self-consistency and TF-IDF retrieval of case-specific labelled examples, individually and in combination (retrieval-augmented order-invariant self-consistency, RAOI-SC), across seven locally served open-weight LLMs on 1,268 Semio2Brain rows. Performance was assessed with weighted- and macro-F1, paired bootstrap confidence intervals, and exact McNemar tests against the self-consistency baseline, and a multi-dimensional reliability profile covering citation coverage and conditional verifiability, retrieval agreement, strict schema adherence, and confidence concentration; a TF-IDF nearest-neighbor classifier and grouped-split sensitivity analyses bounded how much of any gain could reflect retrieval leakage rather than the intervention itself. RAOI-SC had a higher weighted-F1 point estimate than self-consistency for six of seven models, but the improvement was supported by paired confidence intervals for four models and by McNemar testing for only one, Mistral-7B-Instruct-v0.3; the TF-IDF nearest-neighbor baseline alone achieved 55.72 weighted-F1 under row-level splitting, falling to 50.43 and 48.97 once identical-text and publication-reference duplicates were grouped together. These results support a cautious, model-specific reading of retrieval and order permutation as testable prompting interventions rather than a universally superior or clinically validated method, and define a row-level benchmark that does not by itself establish patient- or source-level generalization.

## 1 Introduction

Epilepsy affects an estimated 70 million people worldwide, and roughly one-third of patients continue to experience seizures despite adequate pharmacological treatment (1). For these patients, resective surgery remains the only potentially curative option, and its success depends critically on accurately localizing the seizure onset zone (SOZ) prior to intervention (2). Seizure semiology, the sequence of signs and symptoms a patient exhibits during a seizure, is among the most readily available sources of localizing information, as it can be derived from patient and witness reports without requiring invasive monitoring (3). Epileptologists therefore rely heavily on semiology when formulating a pre-surgical hypothesis about the brain region generating the seizures, particularly in settings where intracranial EEG is unavailable or inconclusive.

SemioLLM (4) established the task and showed that LLM performance depends on prompting strategy. Its observations, however, concern distinct failure modes: confident errors concern calibration; fabricated citations concern factuality; fixed candidate order may induce positional bias; and static few-shot examples may be poorly matched to a query. These mechanisms should not be conflated.

The present interventions target only the latter two mechanisms. Candidate-order permutation tests whether varying the seven-region order stabilizes self-consistency. TF-IDF retrieval replaces static examples with lexically similar labeled rows. Neither intervention directly verifies citations or calibrates probabilities; those properties are evaluated separately.

Both techniques are established inference-time ideas. This study adapts and jointly evaluates them for semiology localization, where outputs are structured seven-region likelihoods and the data are highly imbalanced. Comparisons with earlier models are descriptive because model versions and inference environments differ.

We evaluate the two interventions individually and in combination. RAOI-SC is the combined experimental condition, not a third independent mechanism. Accuracy, attribution, retrieval agreement, format validity, and confidence concentration are reported as separate outcomes.

The contributions are:

- A controlled, paired comparison of candidate-order permutation and TF-IDF-selected examples, individually and combined (RAOI-SC), across seven open-weight LLMs on seizure semiology localization, evaluated against self-consistency with weighted- and macro-F1, exact McNemar tests, and bootstrap confidence intervals rather than point estimates alone.
- A multi-dimensional reliability evaluation that separates citation coverage and conditional verifiability, retrieval agreement, strict schema adherence, and confidence concentration as distinct diagnostic axes instead of a single hallucination score.
- A retrieval leakage audit and TF-IDF nearest-neighbor sensitivity analysis that bounds how far the row-level results can be interpreted as evidence of generalization.

The remainder of this paper is organized as follows. The Related Studies section situates our study within the broader literature on LLMs in clinical decision support, option-order/selection bias, RAG, and hallucination evaluation. The Materials and Methods section details the Semio2Brain dataset, the five baseline prompting strategies reproduced from SemioLLM, our two proposed interventions and their combination, the seven evaluated open-weight models, and the accuracy and hallucination-specific metrics used to assess them. The Results section reports the reproduced baseline results, an ablation isolating the contribution of each intervention, cross-model generalization across general-purpose and biomedical-specialized models, and the associated statistical significance tests. The Discussion section examines the implications of these findings, their limitations, and directions for future studies, and the Conclusion section summarizes the findings.

## 2 Related studies

### 2.1 Large language models for clinical and diagnostic reasoning

General-purpose LLMs have increasingly been evaluated as decision-support tools across medicine, spanning clinical question-answering, differential diagnosis, and report generation. Recent large-scale benchmarks such as MedXpertQA (5), which spans 17 specialties and explicitly targets expert-level reasoning over simple factual recall, illustrate both the rapid pace of this evaluation effort and the extent to which current models still fall short of consistent expert-level performance. This evaluation literature has grown considerably in scope over 2025 and 2026: BRIDGE (6) evaluated 95 LLMs on 87 tasks drawn from real-world clinical data in nine languages and spanning the full patient-care continuum from triage to billing, and found that even the strongest proprietary models remain unreliable on tasks that mirror everyday clinical documentation instead of curated exam questions. A separate strand of this literature has examined whether these capabilities require domain-specific fine-tuning at all. Medprompt (7) showed that a general-purpose foundation model, combined purely with inference-time prompting strategies (namely dynamic few-shot example selection, self-generated CoT, and choice-shuffling ensembling), could match or exceed specialized medical fine-tuning on standard medical benchmarks. More recently, Ray et al. (8) revisited this question specifically for clinical question-answering systems and again found that specialized fine-tuning provided little benefit once comparable inference-time interventions were applied to the general-purpose baseline. Together, these results motivate treating the prompting layer itself, and not the model’s weights, as a primary lever for improving clinical LLM performance. Our proposed interventions build directly on this assumption.

### 2.2 LLM-based seizure semiology localization

Seizure semiology localization is grounded in a substantial clinical evidence base linking specific ictal signs and symptoms to the underlying SOZ (1–3), most recently consolidated in the Semio2Brain database (9), a meta-analysis of surgical outcome case reports that maps free-text semiology descriptions to a region-level likelihood distribution. SemioLLM (4) was the first to cast this mapping as an LLM prompting task, evaluating GPT-3.5, GPT-4, Mixtral-8x7B, and Qwen-72B-Chat under zero-shot, few-shot, CoT, few-shot-CoT, and self-consistency prompting, and reporting weighted-F1 scores that, under the best-performing configuration, approached clinician-level agreement. A complementary line of research has instead pursued fine-tuning: EpiSemoLLM (10) fine-tuned a 7-billion-parameter model directly on curated semiology-outcome pairs and reported regional localization accuracy comparable to that of a panel of epileptologists. These two studies represent the two dominant strategies for adapting LLMs to this task, namely prompting a general-purpose model versus fine-tuning a specialized one, but neither examines whether prompting-only strategies can be further improved without additional training, nor whether the outputs of either approach can be trusted beyond their aggregate accuracy; both gaps are the focus of the present study.

### 2.3 Broader LLM applications in epilepsy and neurology

Beyond semiology-to-region mapping specifically, LLMs have begun to be applied across other epilepsy-related clinical workflows, indicating a rapidly growing niche and not an isolated benchmark. Abeysinghe et al. (11) used pretrained language models to extract structured seizure-frequency information directly from free-text epilepsy evaluation reports, a distinct but related information-extraction problem that, like semiology localization, depends on correctly interpreting unstructured clinical narrative. Zhao et al. (12) instead fine-tuned an LLM, EpilepsyLLM, to represent Japanese-language epilepsy knowledge, extending this line of research to a non-English clinical setting where general-purpose LLMs are typically weaker. Most recently, Li et al. (13) used an LLM as a graph-structure refiner to improve representation learning for EEG-based seizure diagnosis, applying LLMs to a signal-derived, as opposed to text-derived, representation of epilepsy. In a complementary multimodal direction, Zhang et al. (14) introduced Seizure-Semiology-Suite, a large-scale video dataset and benchmark that evaluates multimodal LLMs directly on seizure recordings instead of clinician-written text descriptions, covering perception, temporal sequencing, and narrative report generation. None of these studies, however, addresses the specific combination of concerns raised by SemioLLM, i.e., positional bias in a fixed-region output schema and unverified self-reported reasoning, that motivates our proposed interventions.

### 2.4 Option-order bias and self-consistency in LLM reasoning

Self-consistency (15) improves CoT reasoning by sampling multiple independent reasoning paths at non-zero temperature and aggregating their resulting answers, on the premise that errors in individual reasoning paths are less correlated than the paths’ agreement on a correct answer. However, LLMs evaluated on closed-set, fixed-answer tasks have been repeatedly shown to be sensitive to the order in which the answer options are presented instead of judging each option purely on its merits: Zheng et al. (16) demonstrate across 20 LLMs and three multiple-choice benchmarks that this selection bias stems from a token-level prior over option identifiers (e.g., a preference for the token corresponding to option “A”) that is independent of option content, and propose a label-free debiasing correction. Medprompt (7) instead addressed the same phenomenon at the ensembling stage, shuffling multiple-choice answer options across an otherwise-standard self-consistency loop so that no single ordering can systematically bias the aggregated vote, an intervention that a recent survey of medical-reasoning techniques identified as one of the few training-free methods with a consistent, task-agnostic effect (17). This has remained an active area of research through 2025 and 2026. Guda et al. (18) propose a transferable LoRA fine-tuning approach alongside a more computationally efficient majority-voting scheme across option permutations, reducing the cost of permutation-based debiasing while leaving its underlying need unchanged. Zheng et al. (19) instead attack the same problem at training time, penalizing a model during reinforcement learning whenever its answer is inconsistent across permuted option orderings, a training-time analogue of the inference-time shuffling used here. Working directly in a clinical setting, Lasik et al. (20) reassessed high-performing LLMs on Polish medical licensing exams and found accuracy drops of roughly 28 to 31 percentage points once option-order and guessing artifacts were controlled for, showing that this bias is not merely a general-NLP curiosity but can substantially inflate apparent competence on medical multiple-choice tasks specifically. Tamba (21) further shows, via an exhaustive-permutation diagnostic, that position bias can be masked, not absent, in stronger models, appearing only once accuracy falls into a mid-range band, which cautions against assuming any given model is bias-free without directly testing it. To our knowledge, despite this substantial and growing body of research, option-order permutation has so far only been demonstrated on multiple-choice question answering with a small, per-question answer set, and has not previously been applied to a fixed, larger candidate-region list repeated identically across every query, as required for seizure semiology localization.

### 2.5 Retrieval-augmented in-context example selection

A separate line of research has examined retrieval not as a means of grounding a model’s final answer in external evidence, but as a way of choosing which few-shot examples appear in the prompt, replacing a small, static, hand-picked set with examples dynamically selected for their similarity to the query at hand. Ge et al. (22) applied exactly this idea to biomedical named entity recognition, comparing static few-shot prompting against retrieval-augmented dynamic prompting and finding that TF-IDF-based retrieval of similar examples (the same underlying retrieval mechanism used in this study) consistently outperformed a fixed example set. Outside biomedicine, Trad and Chehab (23) reported a similar pattern for code vulnerability detection, showing that retrieval-augmented few-shot prompting substantially outperformed both random-example few-shot prompting and, notably, fine-tuning on the same task, echoing the fine-tuning-versus-prompting debate discussed above. Both studies support the view that in-context example selection is itself a design choice worth optimizing independently of any downstream retrieval used for factual grounding, but neither has been applied to a structured, fixed-schema clinical prediction task such as seizure semiology localization, where few-shot examples must additionally carry a real, verifiable region-likelihood label rather than a free-text answer.

### 2.6 Retrieval-augmented generation and hallucination mitigation

RAG (24) conditions an LLM’s output on evidence retrieved for the specific input, instead of relying solely on knowledge implicitly encoded in the model’s parameters, and has since been adopted widely across biomedical NLP tasks (25). Its benefit is not merely architectural: in a direct clinical evaluation, Wada et al. (26) compared a locally hosted LLM answering radiology contrast-media consultation questions with and without retrieval augmentation and found that RAG eliminated hallucinated responses entirely across a set of consultation scenarios in which the non-retrieval baseline hallucinated in a non-trivial fraction of cases. Xu et al. (27) reported a similar effect in public-health question answering, where a multi-evidence-guided RAG framework reduced hallucination rates by more than 40% relative to a non-retrieval baseline. This benefit, however, is not universal: Rewthamrongsris et al. (28) compared RAG and non-RAG versions of three proprietary LLMs on infective endocarditis antibiotic-prophylaxis questions and found that retrieval augmentation left one model’s accuracy essentially unchanged while actually reducing the accuracy of the other two. This broader RAG and hallucination literature is also directly relevant to the failure mode SemioLLM itself reported: models fabricating the literature sources cited to justify their reasoning (29), a behavior consistent with broader recent evidence that LLMs fabricate citations even when explicitly asked to justify domain-specific research claims (30). Despite RAG’s demonstrated, if uneven, ability to curb this kind of fabrication elsewhere in biomedicine, prior research on seizure semiology localization has applied it to neither the in-context example selection used for few-shot prompting nor the model’s final predictions, leaving retrieval-based grounding entirely unexplored for this task.

### 2.7 Evaluating hallucination and structured-output reliability

Beyond describing hallucination, parallel studies have developed automatic metrics to measure it. Huang et al. (29) distinguished factuality hallucination, i.e., an output that conflicts with real-world knowledge, from faithfulness hallucination, i.e., an output that is not actually supported by the input or retrieved context provided to the model. This distinction maps directly onto the two kinds of unreliability SemioLLM identified: confidently wrong predictions, and unsupported self-reported citations. For faithfulness specifically, RAGAS (31) and ALCE (32) both operationalized groundedness as agreement between a generated output and the retrieved or cited context it is supposedly based on, decomposing an answer into claims or sentences and checking each against its source; AttrScore (33) and TRUE (34) formalized this same agreement judgment as an attribution or entailment classification problem more broadly. This evaluation landscape has continued to evolve rapidly. FaithBench (35) showed that even the best existing hallucination-detection approaches, including RAGAS-style automated scoring, achieved only near-chance accuracy on the hardest, most borderline hallucinations in summarization. FaithJudge (36) responded by proposing an LLM-as-judge framework built on human-annotated examples with an evolving leaderboard, while the FACTS Grounding leaderboard (37) extended automated grounding evaluation to long-form input at a considerably larger scale than earlier benchmarks. None of these metrics, however, were designed for a task whose output is a fixed-schema numeric distribution over a closed label set, not free text, and none directly measure whether a model’s raw output even conforms to its required output schema. This narrower question, whether a model reliably produces schema-valid structured output at all, is itself an increasingly well-studied failure mode: JSONSchemaBench (38) benchmarked constrained-decoding frameworks across 10,000 real-world JSON schemas, while the Structured Output Benchmark (39) found that near-perfect schema-level compliance can mask substantially lower value-level accuracy. Most relevant to our own setting, Yang et al. (40) directly evaluated schema enforcement and output stability in a clinical note-editing task for locally deployed, open-weight 7-8-billion-parameter models, including Llama3-Med42-8B and Mistral-7B-Instruct-v0.3, and found that prompt-only JSON generation is often insufficient without explicit schema enforcement. Shen (41) reached a similar conclusion for Qwen2.5-7B and Llama-3.1-8B, reporting baseline schema-compliance rates as low as 85.9% that only approached near-100% once a closed-loop validation-repair step is added. This gap between existing free-text faithfulness metrics and our task’s structured-label setting, together with the demonstrated unreliability of prompt-only structured output specifically for the small, open-weight models this study also evaluates, motivates the retrieval-faithfulness and schema-adherence metrics introduced in the Materials and Methods section.

## 3 Materials and methods

We evaluated eight prompting conditions on the 1,268-row Semio2Brain-derived dataset. Table 1 separates the factors changed by each condition. RAOI-SC is explicitly the combination of retrieved examples and candidate-order permutation within a five-sample self-consistency loop.

**Table 1:** Experimental conditions and the factors changed by each condition.

Condition	Fixed examples	Retrieved examples	Reasoning/source	Permutation	Samples	Temperature	Aggregation
Zero-shot	No	No	No	No	1	0	Single output
Few-shot	3 hand-written	No	No	No	1	0	Single output
CoT	No	No	Yes	No	1	0	Single output
Few-shot-CoT	3 hand-written	No	Yes	No	1	0	Single output
SC	No	No	No	No	5	0.7	Median
FS-Retrieved	No	3 real rows	No	No	1	0	Single output
SC-Perm	No	No	No	Yes	5	0.7	Median
RAOI-SC	No	3 real rows	No	Yes	5	0.7	Median

### 3.1 Dataset

We use the Semio2Brain database (9) in this research, which is the same publicly released meta-analysis of surgical case reports on SemioLLM (4), so that our experiments are evaluated against an identical data source. Each row records a free-text seizure semiology description alongside per-patient counts of which of the seven candidate regions (i.e., temporal lobe, frontal lobe, cingulate gyrus, parietal lobe, occipital lobe, insula, and hypothalamus) were implicated by the surgical outcome. Following SemioLLM’s filtering criterion, we retain only rows whose surgical outcome indicates post-operative seizure freedom (Engel class Ia/Ib or ILAE class 1/2) and whose region counts include at least one non-zero value, additionally excluding a small set of semiology categories (Moniz, Psychomotor, Post-ictal Amnesia, and nocturnal) removed on clinician advice in the original research. Table 2 shows one representative row for each of the seven ground-truth regions in the filtered Semio2Brain dataset.

**Table 2:** One representative row per ground-truth region from the filtered Semio2Brain dataset.

ID	Semiology text	Likelihood (%)	GT region
59	Gestural automatisms	Temporal 100	Temporal lobe
81	Aggressive behavior	Frontal 50, Cingulate 50	Frontal lobe
209	Clonic jerks of right arm	Cingulate 50, Parietal 50	Cingulate gyrus
492	Version to the right	Parietal 100	Parietal lobe
191	Stereotyped language	Occipital 100	Occipital lobe
245	Loss of consciousness	Insula 100	Insula
131	Feeling in his head	Hypothalamus 100	Hypothalamus

For every retained row, the per-region counts are normalized into a percentage likelihood distribution summing to 100, and the ground-truth region is taken as the argmax of that distribution. Semiology text is cleaned following the clinician-guided normalization that SemioLLM describes: Excel export artifacts and patient-identifier prefixes are stripped, and a small set of clinical abbreviations is expanded (e.g., “Sz” → “seizure,” “UE” → “upper extremity”).

The class distribution is highly imbalanced; Temporal and Frontal Lobe rows constitute more than 86% of the dataset. We therefore report both weighted-F1 and macro-F1, together with class-specific results and paired accuracy analyses. Table 3 summarizes the class distribution.

**Table 3:** Class distribution of the filtered Semio2Brain dataset.

Region	*N*	% of dataset
Temporal lobe	705	55.60
Frontal lobe	394	31.07
Parietal lobe	63	4.97
Occipital lobe	49	3.86
Cingulate gyrus	37	2.92
Hypothalamus	11	0.87
Insula	9	0.71
Total	1,268	100.00

### 3.2 Baseline prompting strategies

All conditions used the same persona, clinical query, seven candidate regions, and JSON-like likelihood schema. Single-completion conditions used temperature 0. Self-consistency conditions generated five samples at temperature 0.7 and aggregated valid seven-region distributions by componentwise median followed by normalization.

Table 1 specifies whether each condition includes fixed examples, retrieved examples, reasoning/source fields, candidate-order permutation, repeated sampling, temperature, and aggregation.

### 3.3 Proposed methods

Figure 1 summarizes the shared pipeline and the two experimental factors.

**Figure 1:**
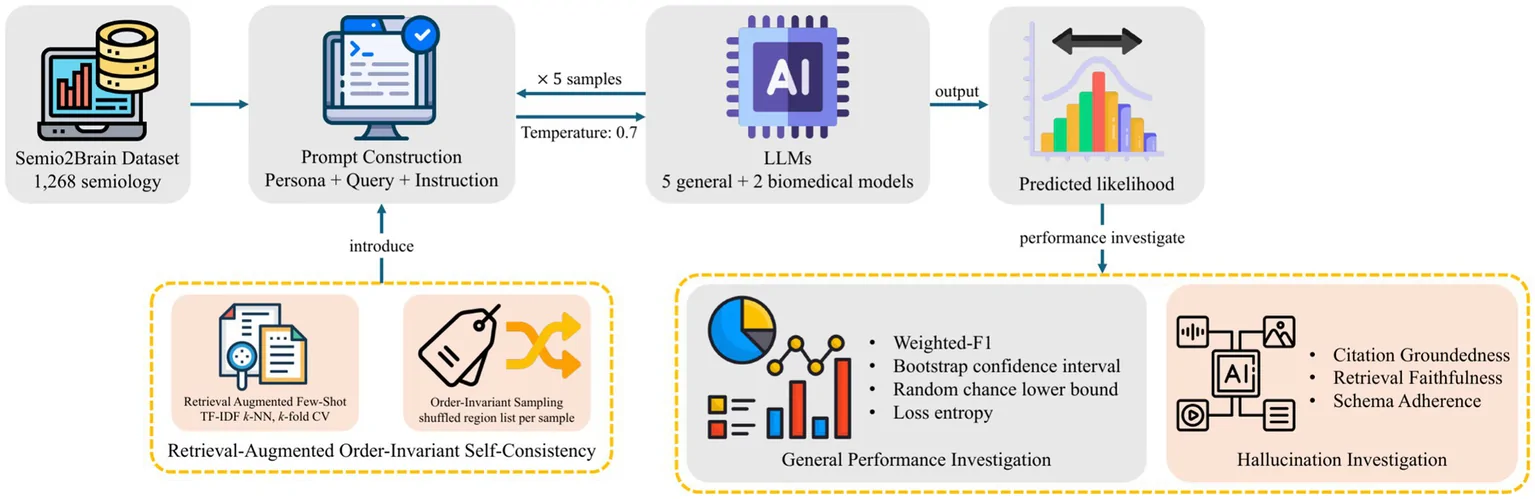
Experimental pipeline separating retrieval-selected examples from candidate-order permutation.

The interventions address different hypotheses. SC-Perm changes only the order of candidate regions across the five samples. FS-Retrieved supplies three TF-IDF nearest labeled rows in a single-completion prompt. RAOI-SC uses those same three retrieved examples inside the five-sample permuted loop.

#### 3.3.1 Experimental conditions

For each held-out query, TF-IDF cosine similarity was computed against the other four folds, and the top three rows were formatted as worked examples. This prevents a row from retrieving itself but does not guarantee independence of patients, duplicated descriptions, or publication sources.

Candidate-order permutation independently shuffled all seven region names for each SC-Perm or RAOI-SC sample. The same order was used consistently in the query, schema, and worked-example likelihood object, after which parsed values were restored to canonical order.

The five valid likelihood vectors were combined by componentwise median and renormalized. Rows with no valid sample followed the prespecified fallback and were counted as fully invalid in the strict-schema analysis.

The leakage audit found 154 repeated-text groups covering 471 rows, including 66 groups with conflicting labels. Patient identifiers are unavailable. We therefore report additional identical-text-grouped and publication-reference-grouped retrieval sensitivity analyses.

Complete pseudocode and verbatim prompts are provided in Supplementary material.

### 3.4 Large language models

Seven open-weight instruction-tuned models (7–14 billion parameters) were served on the supercomputer, one NVIDIA H100 80-GB GPU per job. The server used vLLM 0.27.0, PyTorch 2.13.0 + cu130, Transformers 5.15.0, automatic/bfloat16 dtype, tensor-parallel size 1, maximum context 8,192, and maximum completion length 4,096. Client analysis used Python 3.9.25, NumPy 2.0.2, pandas 2.3.3, SciPy 1.13.1, and scikit-learn 1.6.1. Fold creation, subsampling, and candidate permutations used seed 0; no explicit model-side seed or top-p was passed. Prompts were assembled programmatically and were not manually truncated. Exact model commit hashes are listed in Supplementary material. Table 4 lists the evaluated models, parameter counts, and Hugging Face repositories.

**Table 4:** The seven open-weight models evaluated in this study.

Model	Params	Hugging Face repository
General LLMs		
Qwen2.5-14B-Instruct	14B	Qwen/Qwen2.5-14B-Instruct
Mistral-7B-Instruct-v0.3	7B	mistralai/Mistral-7B-Instruct-v0.3
Gemma-2-9B-it	9B	google/gemma-2-9b-it
Phi-4	14B	microsoft/phi-4
GLM-4-9B-Chat	9B	zai-org/glm-4-9b-chat-hf
Biomedical-specialized LLMs		
OpenBioLLM-8B	8B	aaditya/Llama3-OpenBioLLM-8B
Med42-v2-8B	8B	m42-health/Llama3-Med42-8B

### 3.5 Evaluation metrics and statistical analysis

Primary outcomes were weighted-F1 and macro-F1 over the seven argmax labels. We also report accuracy because McNemar’s test operates on paired correctness. Scores are percentages unless stated otherwise.

For marginal uncertainty, 2,000 standard bootstrap samples of *N* = 1,268 rows were drawn with replacement, and 95% percentile confidence intervals were computed. For SC versus RAOI-SC, row indices were resampled jointly, and confidence intervals were calculated for paired weighted- and macro-F1 differences. Exact two-sided McNemar tests used discordant paired correctness outcomes.

Citation reporting was decomposed into coverage (a non-empty source was supplied) and conditional verifiability (a supplied source matched the curated bibliography). No numerical citation-similarity threshold was used. Retrieval faithfulness measured cosine agreement with the retrieved examples’ label consensus and does not measure factual correctness.

Strict schema adherence required a parseable seven-region object containing every required key and numeric, finite, non-negative values. Fully invalid rows had no valid sample among five generations. Loss entropy quantified concentration of the predicted distribution; it was not interpreted as calibration or overconfidence.

A TF-IDF nearest-neighbor label-consensus classifier was evaluated under the original row folds, identical-text grouping, and publication-reference grouping. Reference groups were reconstructed by forward-filling the source spreadsheet’s reference cells; this sensitivity analysis is not a patient-level validation.

## 4 Results

### 4.1 Baseline comparison

Table 5 reports weighted-/macro-F1 (W/M) for the seven locally evaluated models; published SemioLLM comparator rows retain weighted-F1 only. Figure 2 shows standard bootstrap 95% confidence intervals. Cross-study comparisons are descriptive and are not used as formal evidence of superiority.

**Table 5:** Performance across prompting conditions.

Model	ZS (W/M)	FS (W/M)	CoT (W/M)	FS-CoT (W/M)	SC (W/M)	RAOI-SC (W/M)
SemioLLM (4) (published values from Figure 2a and Supplementary Data of ref.4)						
GPT-3.5	38.17	48.05	49.08	51.46	50.99	–
GPT-4.0	52.27	50.78	52.30	51.72	53.44	–
Mixtral-8x7B	51.66	45.41	52.76	52.64	50.45	–
Qwen-72B	39.21	44.31	45.41	47.91	45.70	–
LlaMa2-70B	38.54	39.78	42.06	39.92	41.57	–
LlaMa3-70B	39.84	50.06	49.21	48.03	48.65	–
OpenBioLLM-70B	38.56	46.63	52.19	50.01	53.06	–
Med42-70B	45.10	31.09	46.70	45.05	47.63	–
Clinician 1	48.77	–	–	–	–	–
Clinician 2	46.75	–	–	–	–	–
This research						
Qwen2.5-14B-Instruct	51.93/21.15	49.05/21.63	52.24/22.22	50.69/22.87	51.70/21.37	55.08/21.47
Mistral-7B-Instruct-v0.3	37.36/11.58	42.98/18.23	49.84/20.20	48.25/23.34	39.61/12.49	54.35/21.16
Gemma-2-9B-it	44.38/16.39	44.67/18.16	48.62/20.95	47.15/22.61	43.38/15.39	51.93/23.04
Phi-4	53.55/24.32	47.57/22.05	51.11/24.19	42.48/21.16	52.52/23.84	54.15/21.83
GLM-4-9B-Chat	39.73/10.21	39.73/10.21	41.66/12.44	45.82/17.66	39.50/10.15	45.78/14.16
OpenBioLLM-8B	20.59/9.76	38.16/13.88	44.35/17.26	32.02/13.13	41.26/11.79	37.68/14.39
Med42-v2-8B	44.63/18.97	35.42/13.52	48.73/20.16	37.54/14.17	46.82/19.85	48.93/19.92

Locally evaluated models are weighted-/macro-F1 (W/M, %); published SemioLLM comparator rows report weighted-F1 only.

**Figure 2:**
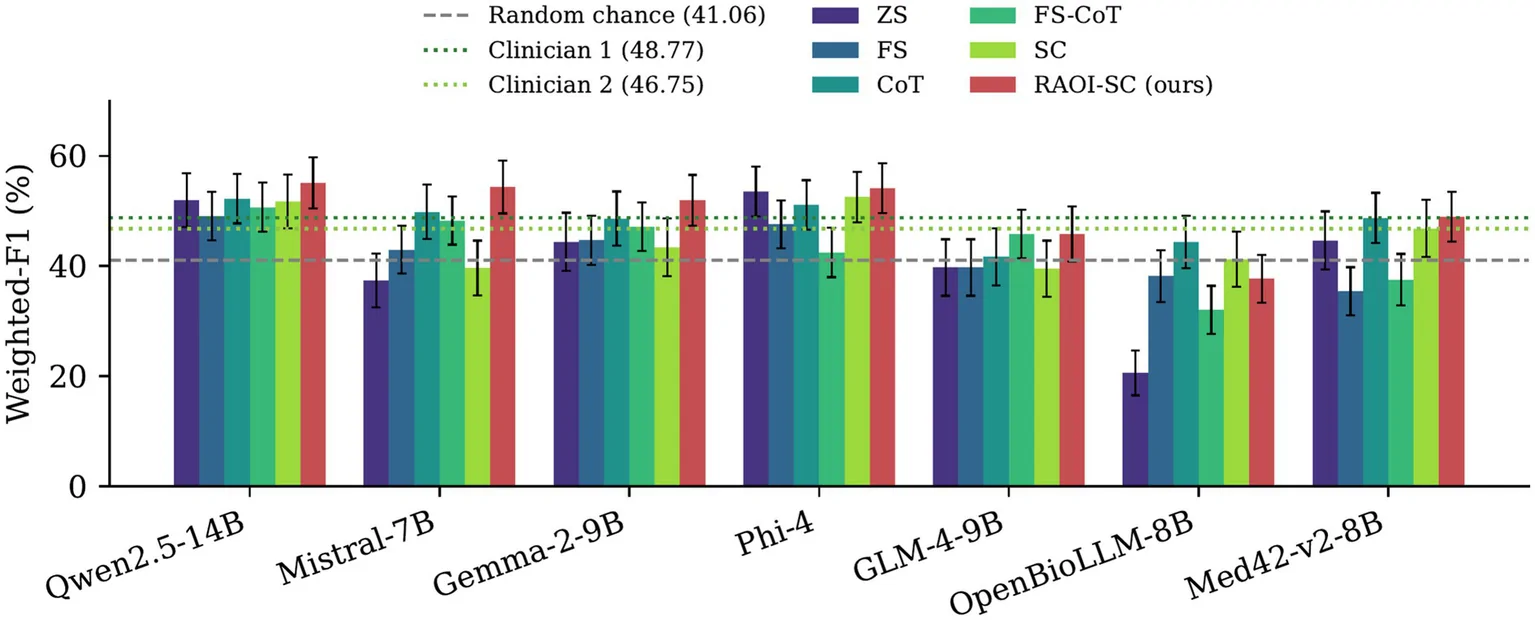
Weighted-F1 for baseline conditions and RAOI-SC. Error bars are 95% percentile confidence intervals from 2,000 standard bootstrap samples of all N rows.

Across local models, weighted- and macro-F1 often ranked conditions differently. Rare-region performance remained low, and aggregate weighted-F1 was driven mainly by temporal and frontal lobe rows. Accordingly, apparent gains should not be equated with balanced anatomical localization.

Figures 3–5 provide descriptive heatmap, parameter-count, and class-specific views. Differences from previously published models may reflect model versions, inference stacks, and study dates as well as model capability.

**Figure 3:**
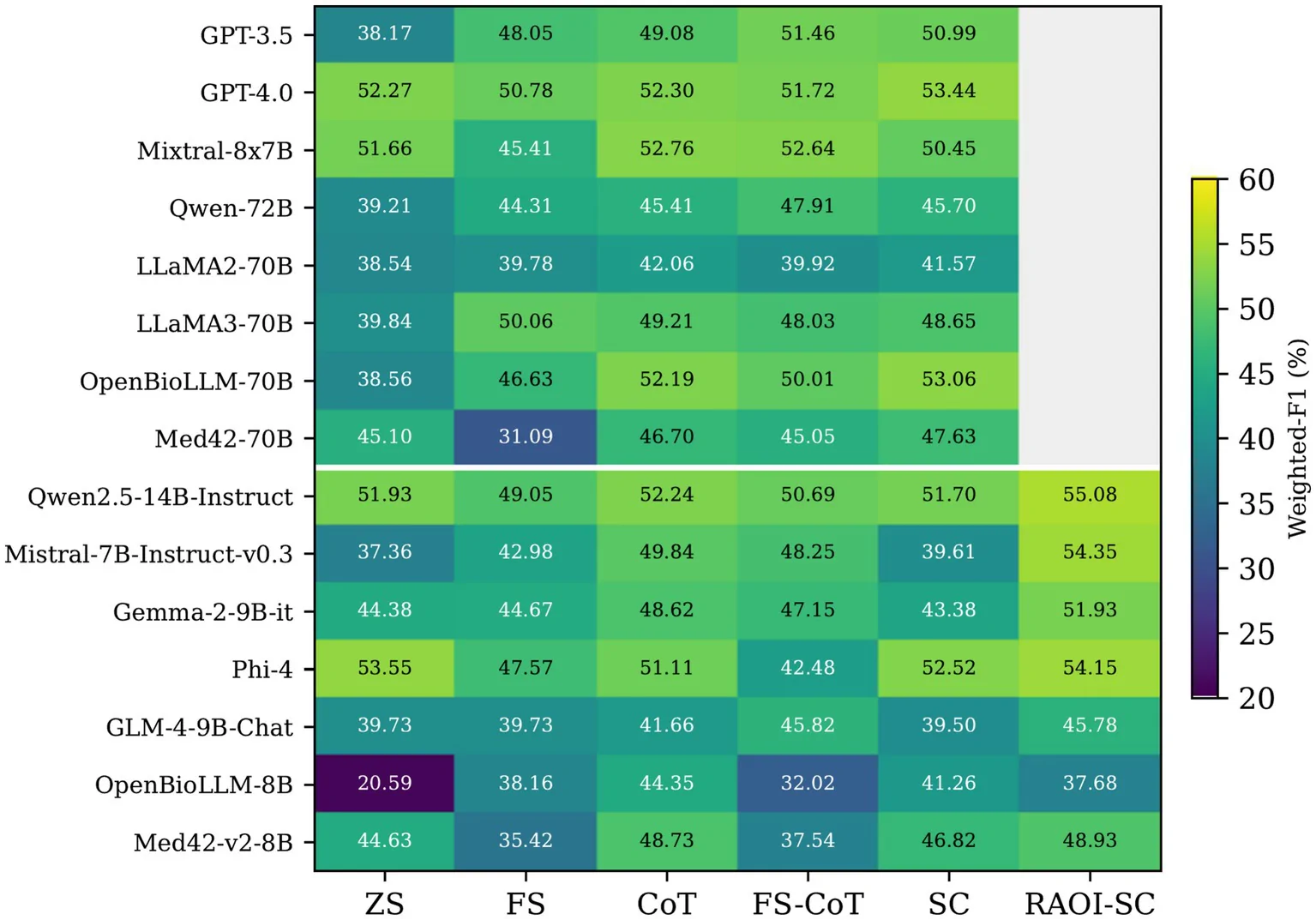
Heatmap of weighted-F1 (%) for every model in Table 5 across the five baseline strategies and RAOI-SC. Top block: SemioLLM’s published models. Bottom block: our experiments.

**Figure 4:**
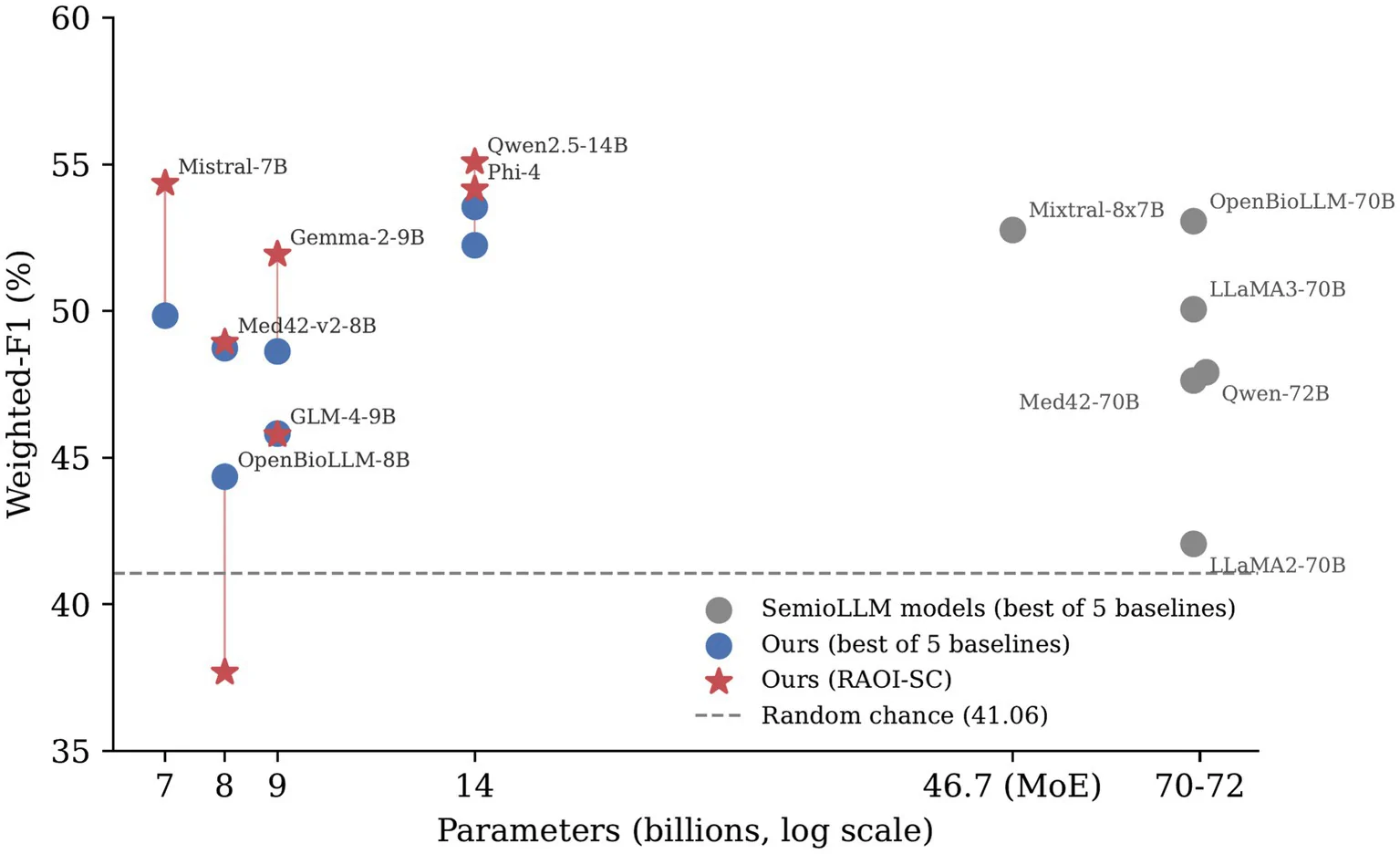
Best-of-5-baseline-strategies weighted-F1 (%) versus parameter count (log scale) for every model in Table 5 with a disclosed parameter count. Gray circles: SemioLLM’s published models. Blue circles: our models’ best baseline strategy. Red stars: our models’ RAOI-SC score, connected to the corresponding blue circle to show the lift from our proposal.

**Figure 5:**
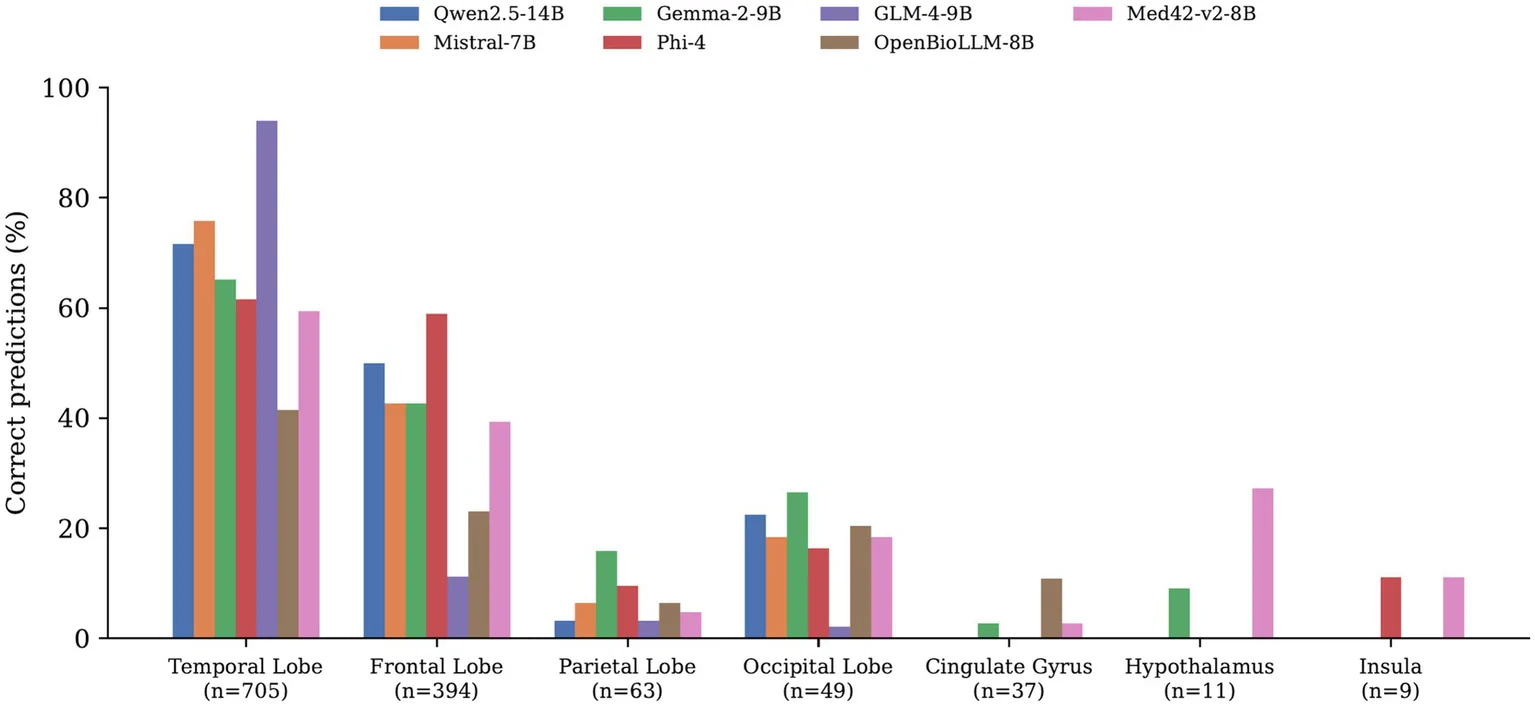
Proportion of RAOI-SC predictions that correctly match the ground-truth region. Regions are ordered by dataset prevalence, with the per-region sample count *n* (from Table 3) shown on the *x*-axis.

Class-specific analysis confirmed near-zero accuracy for many model–region combinations among cingulate gyrus, hypothalamus, and insula. This ceiling is clinically important and is not captured by the weighted-F1 headline.

### 4.2 Ablation study

Table 6 compares SC with three conditions defined in Table 1. FS-Retrieved is a single deterministic completion using retrieved examples, SC-Perm is the five-sample, temperature-0.7 self-consistency loop with candidate order permuted, and RAOI-SC combines retrieval with that permuted self-consistency loop. Because FS-Retrieved and the two self-consistency-based conditions differ in sampling as well as in the factor of interest, this comparison is descriptive rather than a clean factorial decomposition; it nonetheless prevents interpreting RAOI-SC as an independent third mechanism separate from its two components.

**Table 6:** Ablation conditions reported as weighted-/macro-F1 (W/M, %).

Model	SC (W/M)	FS-retrieved (W/M)	SC-Perm (W/M)	RAOI-SC (W/M)
Qwen2.5-14B-Instruct	51.70/21.37	55.10/23.40	52.48/24.00	55.08/21.47
Mistral-7B-Instruct-v0.3	39.61/12.49	53.58/20.85	43.98/14.99	54.35/21.16
Gemma-2-9B-it	43.38/15.39	53.39/18.01	44.66/20.65	51.93/23.04
Phi-4	52.52/23.84	53.01/22.26	41.69/20.79	54.15/21.83
GLM-4-9B-Chat	39.50/10.15	40.63/13.13	40.00/10.94	45.78/14.16
OpenBioLLM-8B	41.26/11.79	34.17/13.56	41.17/11.94	37.68/14.39
Med42-v2-8B	46.82/19.85	49.11/18.37	46.15/19.37	48.93/19.92

RAOI-SC had a higher weighted-F1 point estimate than SC for six models, but macro-F1 changes were less uniform. Retrieval produced most of the weighted-F1 change for several models, although the contrast also changes example presence, provenance, and relevance and therefore does not isolate nearest-neighbor selection. Figures 6–8 visualize the ablation results across models and anatomical regions.

**Figure 6:**
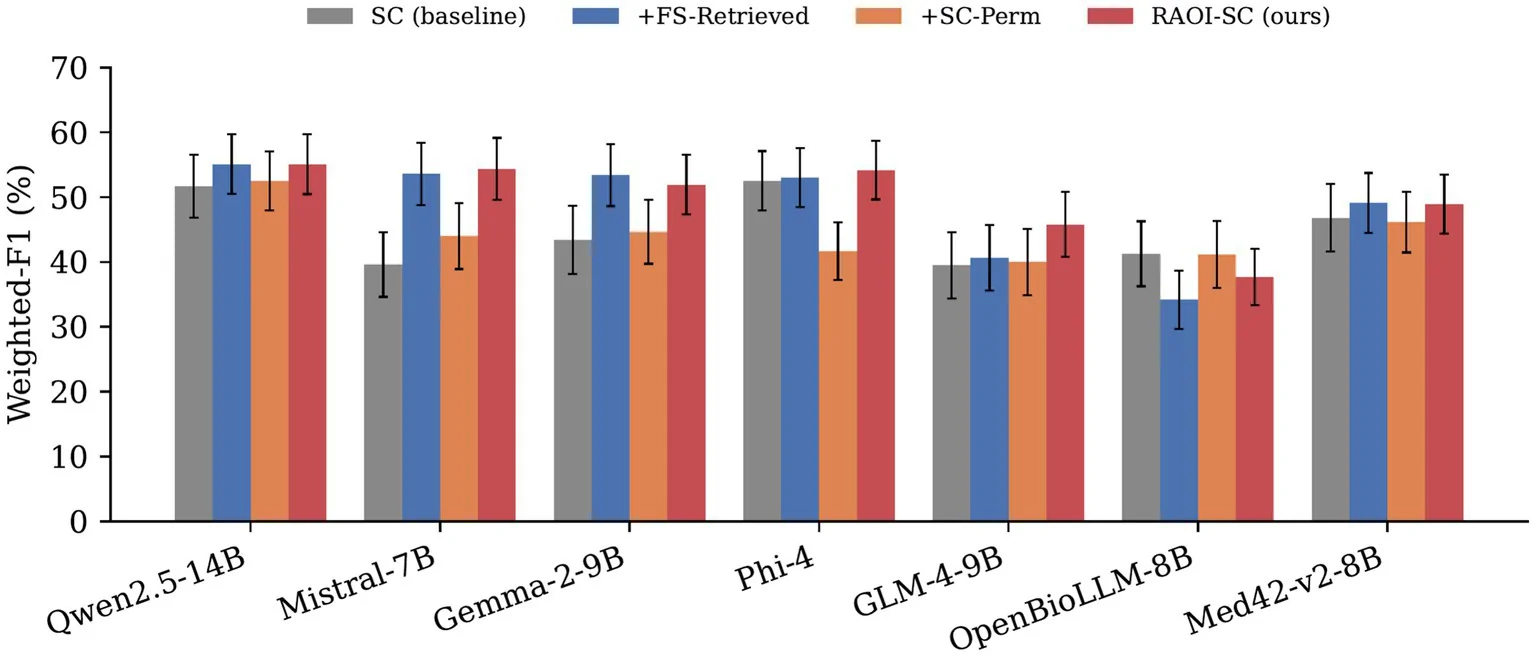
Ablation of retrieval and candidate-order permutation. Error bars are 95% percentile confidence intervals from 2,000 standard bootstrap samples of all N rows.

**Figure 7:**
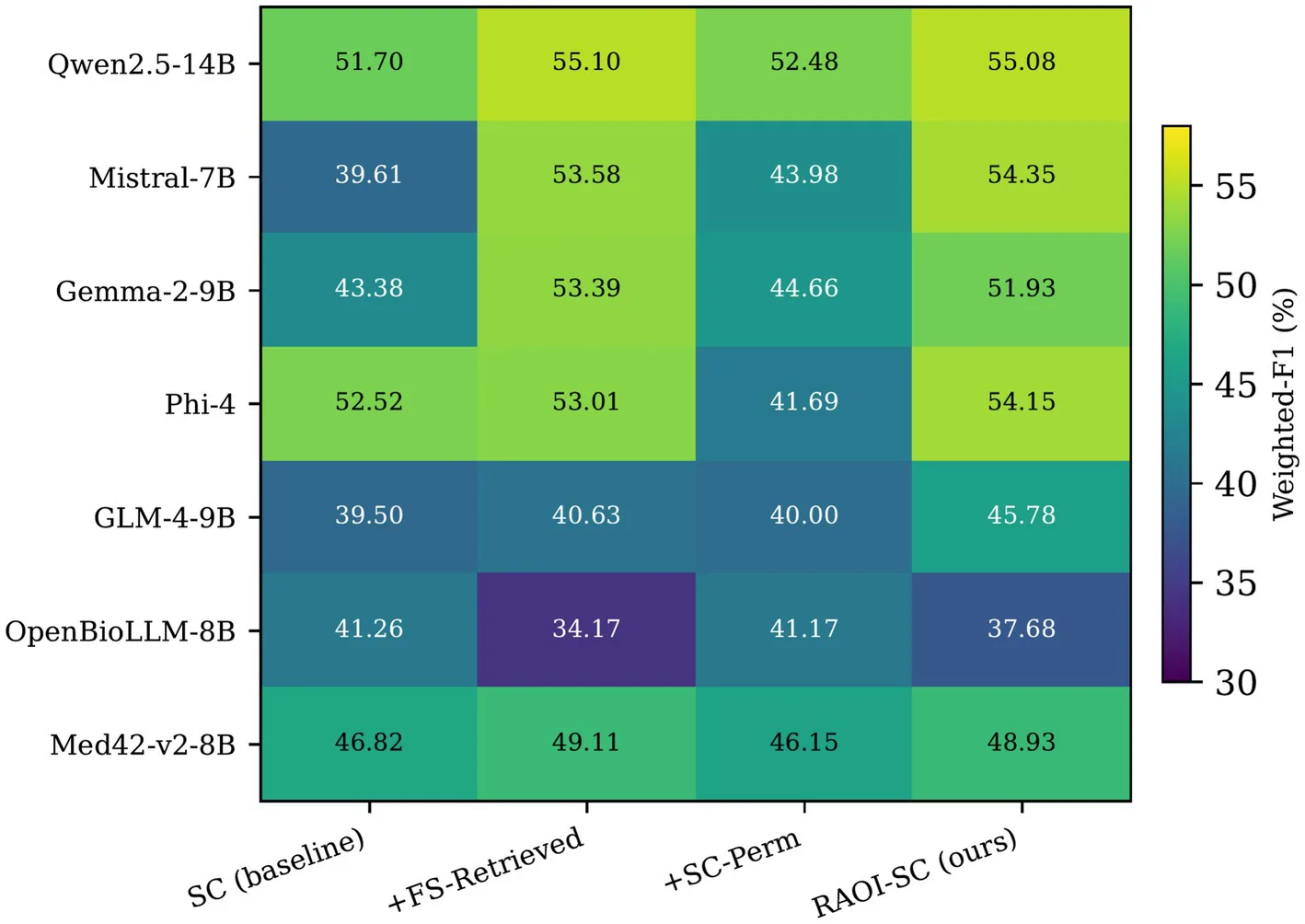
Heatmap of weighted-F1 (%) for every model in Table 6 across the four ablation conditions.

**Figure 8:**
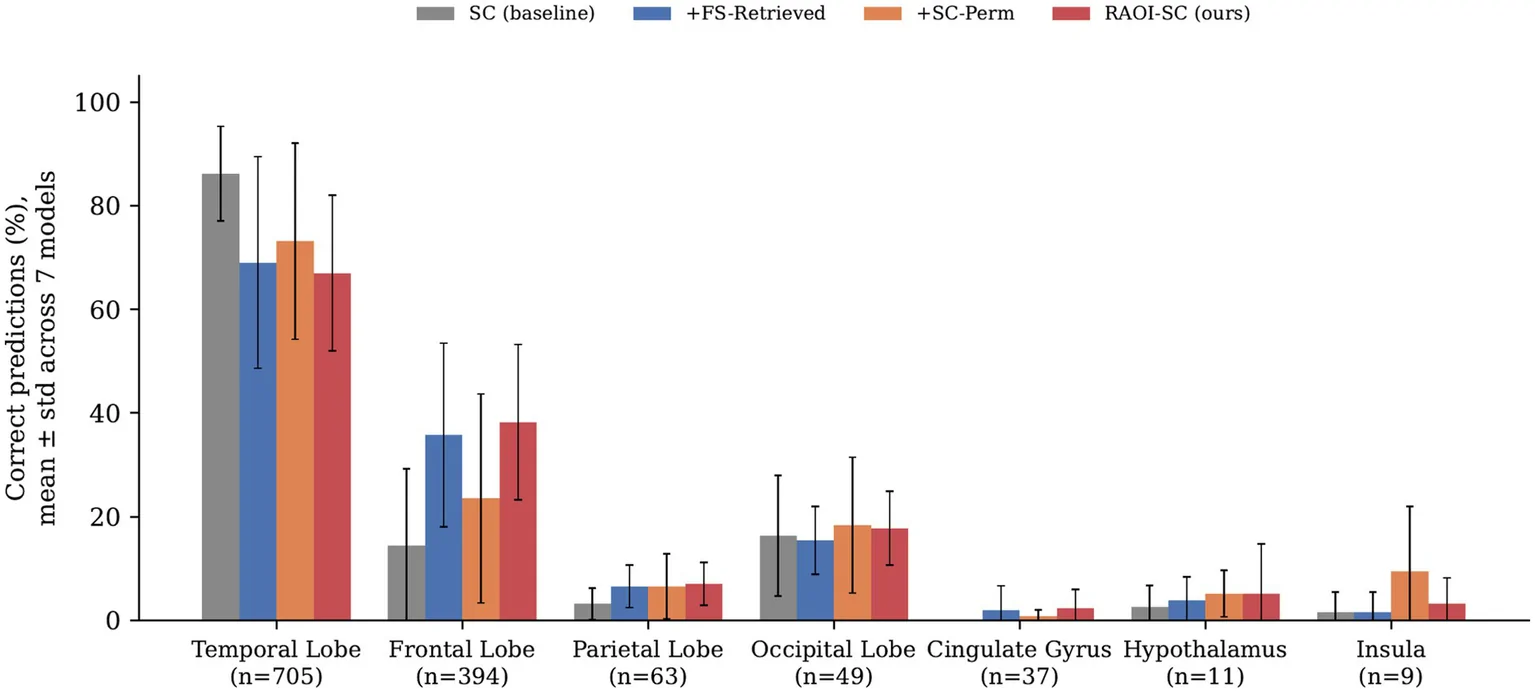
Proportion of predictions that correctly match the ground-truth region for each of the four ablation conditions, mean ± one standard deviation across all seven models. Regions are ordered by dataset prevalence, with the per-region sample count *n*, taken from Table 3, shown on the *x*-axis.

### 4.3 Cross-model reliability diagnostics

The seven models form a small heterogeneous sample and do not support population-level claims about model families. Table 7 therefore presents model-specific diagnostics.

**Table 7:** Model-specific reliability diagnostics.

Model	Cite coverage	Cite verif.	Faith. FSR	Faith. RAOI	Strict SC	Strict RAOI	Invalid RAOI	Entropy SC/RAOI
Qwen2.5-14B	100.0%	42.9%	0.729	0.764	99.72%	97.70%	0.32%	0.351/0.876
Mistral-7B	42.4%	82.2%	0.701	0.741	70.66%	83.61%	0.47%	0.420/0.774
Gemma-2-9B	79.1%	3.0%	0.823	0.790	99.95%	99.95%	0.00%	0.433/0.595
Phi-4	99.9%	48.4%	0.735	0.753	99.92%	99.94%	0.00%	0.588/0.810
GLM-4-9B	95.4%	24.4%	0.215	0.188	45.00%	41.04%	16.32%	0.768/0.942
OpenBioLLM-8B	13.0%	93.0%	0.410	0.390	81.58%	36.07%	12.54%	0.545/0.980
Med42-v2-8B	22.0%	92.8%	0.642	0.615	97.48%	72.46%	1.26%	0.561/0.941

Citation verifiability is conditional on citation coverage; faithfulness is retrieval-label agreement; entropy is confidence concentration, not calibration.

Citation coverage ranged from 13.0 to 100.0%. High conditional verifiability for OpenBioLLM and Med42 applied only to the minority of outputs that supplied a source, whereas Qwen and Phi supplied sources nearly always but fewer than half matched the curated bibliography.

Retrieval faithfulness changed little between FS-Retrieved and RAOI-SC and describes agreement with retrieved labels, not correctness. Strict validation revealed substantial fully invalid RAOI-SC rows for GLM-4 (16.32%) and OpenBioLLM (12.54%). Entropy is reported only as confidence concentration.

Taken together, the diagnostics show that task accuracy, attribution, format validity, and concentration are distinct properties and should not be collapsed into a single hallucination score. Figure 9 summarizes these reliability diagnostics.

**Figure 9:**
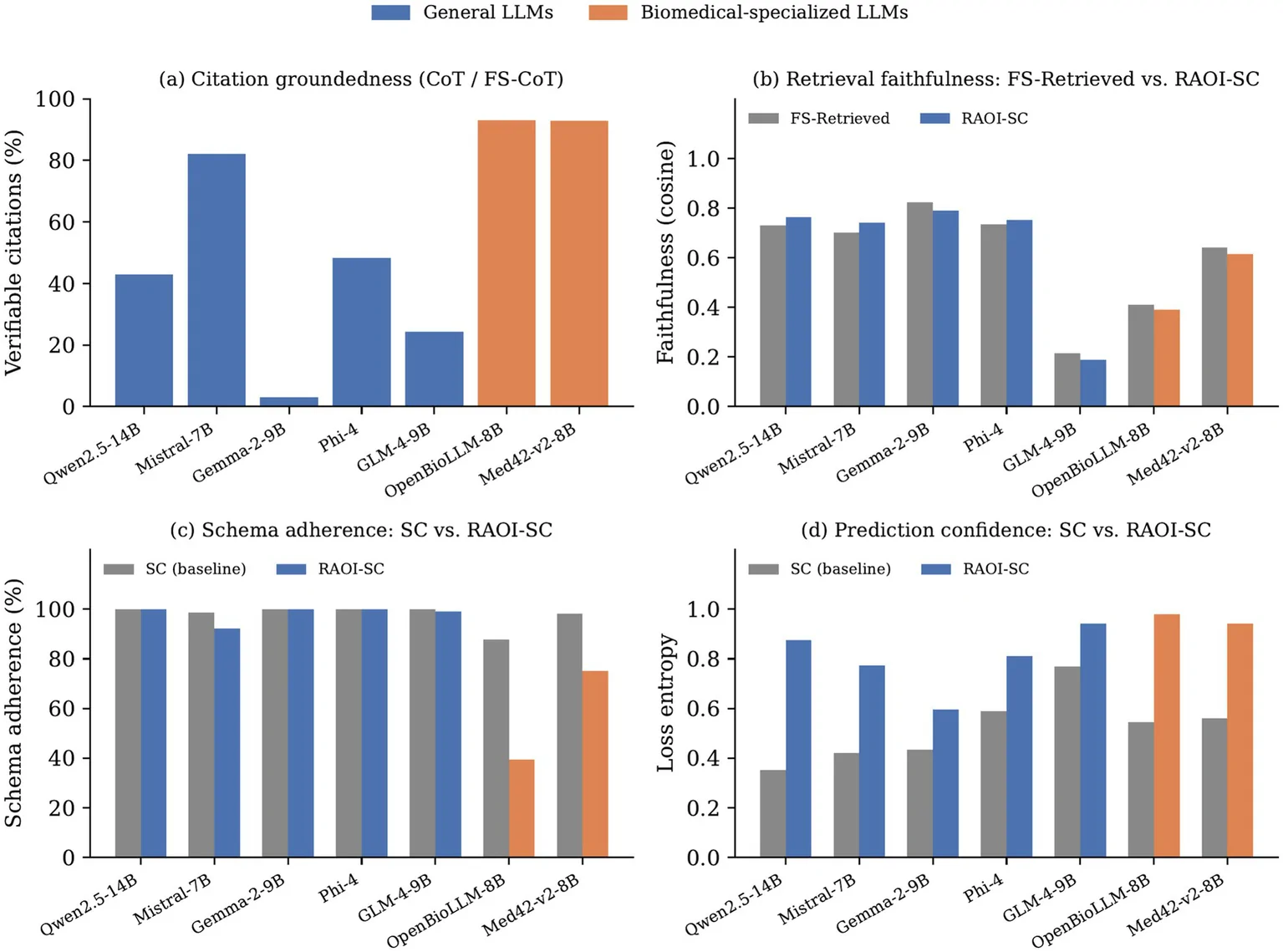
Descriptive reliability diagnostics. **(a)** Conditional citation verifiability; **(b)** retrieval faithfulness (agreement with retrieved labels); **(c)** permissive-parsing schema adherence (strict values in Table 7); **(d)** loss entropy (confidence concentration, not calibration).

### 4.4 Paired statistical comparison and retrieval sensitivity

Table 8 reports RAOI-SC minus SC using 2,000 paired all-row bootstrap resamples and exact two-sided McNemar tests. Weighted-F1 intervals excluded zero positively for Qwen, Mistral, Gemma, and GLM-4; Phi and Med42 were inconclusive; OpenBioLLM decreased. Only Mistral showed a significant accuracy increase, while OpenBioLLM and Med42 had significant accuracy decreases.

**Table 8:** Paired RAOI-SC minus SC comparisons on the same 1,268 rows.

Model	Δ weighted-F1 (95% CI)	Δ macro-F1 (95% CI)	Δ accuracy	McNemar p
Qwen2.5-14B	+3.38 [0.65, 6.09]	+0.09 [−1.65, 1.68]	−0.16	0.954
Mistral-7B	+14.74 [11.53, 17.82]	+8.66 [6.08, 11.23]	+8.12	4.27 × 10⁻⁷
Gemma-2-9B	+8.55 [5.32, 11.81]	+7.65 [5.04, 10.47]	−3.15	0.0557
Phi-4	+1.63 [−1.20, 4.41]	−2.01 [−5.06, 0.77]	+0.63	0.711
GLM-4-9B	+6.28 [4.25, 8.15]	+4.01 [2.37, 5.71]	+1.03	0.223
OpenBioLLM-8B	−3.58 [−7.31, −0.05]	+2.60 [0.51, 4.55]	−18.14	4.90 × 10⁻^2^⁴
Med42-v2-8B	+2.11 [−1.15, 5.44]	+0.07 [−4.25, 3.60]	−8.75	6.04 × 10⁻⁸

Confidence intervals use 2,000 paired all-row bootstrap resamples; *p*-values are exact two-sided McNemar tests.

Table 9 reports the retrieval leakage sensitivity analysis. The TF-IDF label-consensus baseline achieved 55.72 weighted-F1 under row-held-out folds, 50.43 with identical-text grouping, and 48.97 with publication-reference grouping. These declines bound interpretation of the LLM retrieval conditions, which used the same row-level folds.

**Table 9:** TF-IDF nearest-neighbor label-consensus sensitivity analysis (%).

Split definition	Weighted-F1	Macro-F1	Accuracy
Original row-held-out folds	55.72	21.58	58.04
Identical-text grouped	50.43	20.69	51.10
Publication-reference grouped	48.97	19.34	49.68

The analyses support model- and metric-specific conclusions rather than a universal RAOI-SC benefit.

## 5 Discussion

### 5.1 Principal findings

RAOI-SC changed performance in a strongly model- and metric-dependent manner. Four models had paired weighted-F1 intervals excluding zero in the positive direction, but only Mistral had a significant accuracy improvement by McNemar testing. Retrieval was the larger contributor for several models, while order permutation alone was smaller and inconsistent.

The nearest-neighbor baseline and grouped-split analyses materially qualify the result. TF-IDF label consensus alone was competitive under row-level splitting, and performance fell when identical descriptions or publication sources were kept together. The LLM results therefore measure benchmark-row performance, not unseen-patient or unseen-source generalization.

Reliability measures exposed separate trade-offs: conditional citation verifiability depended on coverage; retrieval faithfulness could reward propagation of a wrong neighbor; strict schema validation identified failures hidden by permissive parsing; and entropy did not establish calibration.

The rarest regions remained poorly recognized. Neither aggregate F1 nor these retrospective diagnostics demonstrate clinical benefit, diagnostic safety, or readiness for patient care.

### 5.2 Relation to prior studies

The findings are consistent with prior reports that example selection and candidate order affect inference-time performance. Our evidence does not establish novelty beyond adapting and jointly evaluating these established techniques in this benchmark. Comparisons with older, larger, or proprietary models remain descriptive because experimental conditions differ.

### 5.3 Limitations

The study has several limitations:

- The primary folds hold out rows, not patients or publications; identifiers are unavailable and repeated descriptions cross folds.
- A further limitation is that the current experiments did not compare fixed, random, and nearest real examples under otherwise identical prompts; retrieval effects therefore cannot be attributed solely to neighbor selection.
- TF-IDF is lexical; the data are highly imbalanced and single-label; rare-region macro estimates are unstable; and no external cohort or clinician error adjudication was performed.
- Citation verifiability is not a measure of completeness, retrieval agreement is not correctness, schema adherence is not factuality, and entropy is not calibration. Bootstrap intervals reflect row sampling, not repeated model-generation uncertainty. The number of folds, retrieved examples, self-consistency samples, temperature, and resampling count were fixed for the completed HPC experiment rather than systematically tuned, so their sensitivity remains untested. The seven open-weight models evaluated in the completed run do not represent the full set of models available in 2026.

### 5.4 Future studies

Future studies should use patient- or source-grouped external cohorts; compare fixed, random, and nearest real examples under identical prompts; repeat generation across seeds; evaluate dense clinical retrievers; report calibrated probabilities and class-specific results; and include blinded clinician adjudication.

## 6 Conclusion

In this row-level Semio2Brain benchmark, combining TF-IDF-selected examples with candidate-order-permuted self-consistency produced model- and metric-dependent effects. Four models had positive paired weighted-F1 intervals, but only Mistral had a significant paired accuracy improvement; OpenBioLLM and Med42 showed significant accuracy decreases. Grouped retrieval sensitivity and strict schema validation further weaken broad performance claims. RAOI-SC should therefore be viewed as a testable prompting configuration, not a generally superior or clinically validated method. The study’s practical contribution is a transparent comparison framework incorporating paired analyses, macro-F1, leakage sensitivity, citation coverage, strict schema validation, retrieval agreement, and confidence concentration for future patient-level evaluation.

## Data Availability

The Semio2Brain database analyzed in this study is publicly available at https://github.com/thenineteen/Semio2Brain-Database. The code and derived results are available at https://zenodo.org/records/22247634.
