# Supplementary material for: Toward trustworthy diagnostic AI: debiasing and grounding large language models for seizure semiology interpretation
Source: Front Neurol. 2026 Oct 29;17:1990185. doi: 10.3389/fneur.2026.1990185 (PMC13754667; doi:10.3389/fneur.2026.1990185)
Supplement: Supplementary file 1 [file Data_Sheet_1.pdf]

Supplementary Material

Toward Trustworthy Diagnostic AI: Debiasing and Grounding Large Language Models for Seizure Semiology Interpretation

This supplement provides the complete, verbatim prompt templates and worked few-shot examples used to query every language model evaluated in the main text, for all eight prompting conditions (zero-shot, few-shot, CoT, few-shot-CoT, self-consistency, self-consistency-perm, few-shot-retrieved, and RAOI-SC). All templates and examples are also available as executable source code in the accompanying code repository (see Data Availability Statement in the main text).

S1 Prompt Structure Overview

Every query sent to a model is assembled from up to four parts, in this order: (1) a fixed persona, (2) worked few-shot examples (few-shot strategies only), (3) the query itself, and (4) a schema instruction. Table S1 summarizes which parts are used by each strategy.

Strategy	Few-shot examples	Reasoning + Source fields	Example source
Zero-shot	No	No	–
Few-shot	Yes ($k = 3$, fixed)	No	Hand-written (below)
CoT	No	Yes	–
Few-shot-CoT	Yes ($k = 3$, fixed)	Yes	Hand-written (below)
Self-consistency	No (5× zero-shot)	No	–
Self-consistency-perm (“SC-Perm” in main text)	No (5× zero-shot, permuted regions)	No	–
Few-shot-retrieved (“FS-Retrieved”/“FSR” in main text)	Yes ($k = 3$, retrieved per row)	No	Nearest neighbors from held-out pool
RAOI-SC	Yes ($k = 3$, retrieved per row)	No	Nearest neighbors, 5× permuted

Table S1: Prompt components used by each of the eight evaluated strategies.

S1.1 Persona (fixed, all strategies)

```
You are a knowledgeable assistant specializing in epilepsy with 30 years of
↪ experience. You are able to answer questions about the brain regions associated
↪ with different semiologies and lateralization such that post operative seizure
↪ freedom is achieved.
```

S1.2 Query Template

```
A patient comes into the clinic showing a semiological behaviour '{semiology_text}'.
↪ Determine the likelihood of a percentage of Seizure Onset Zones (SOZ) involved
↪ for the given list {regions}, such that postoperative seizure freedom is
↪ obtained.
```

Here, `{semiology_text}` is the free-text semiology description for the case being queried, and

{regions} is the list of seven candidate brain regions (Temporal Lobe, Frontal Lobe, Cingulate Gyrus, Parietal Lobe, Occipital Lobe, Insula, Hypothalamus), in canonical order for all strategies except the permutation-debiased ones, where a freshly shuffled order is substituted on every resampled reasoning path (see main text Section 3.3.1, "Experimental conditions").

S1.3 Schema Instruction

Zero-shot / few-shot / self-consistency / retrieved variants:

```
The following is the schema you must follow:
------------------
SCHEMA: The output should be formatted as a JSON object.
------------------
'Likelihood': {"Temporal Lobe": "x%", "Frontal Lobe": "x%", "Cingulate Gyrus": "x%",
↪  "Parietal Lobe": "x%", "Occipital Lobe": "x%", "Insula": "x%", "Hypothalamus":
↪  "x%"} -- one entry per region.
Please answer in an unbiased manner. When you do not have information about a brain
↪  region or semiology category, report it as '0%' rather than omitting it. Do not
↪  add any keys beyond the ones specified above.
```

CoT / few-shot-CoT (adds two fields ahead of Likelihood):

```
The following is the schema you must follow:
------------------
SCHEMA: The output should be formatted as a JSON object.
------------------
'Reasoning': break the question down and solve it step by step with explained
↪  reasoning.
'Source': cite the source used for reasoning.
'Likelihood': {"Temporal Lobe": "x%", "Frontal Lobe": "x%", "Cingulate Gyrus": "x%",
↪  "Parietal Lobe": "x%", "Occipital Lobe": "x%", "Insula": "x%", "Hypothalamus":
↪  "x%"} -- one entry per region.
Please answer in an unbiased manner. When you do not have information about a brain
↪  region or semiology category, report it as '0%' rather than omitting it. Do not
↪  add any keys beyond the ones specified above.
```

S1.4 Worked Example Format (few-shot / few-shot-CoT / few-shot-retrieved)

Each worked example is rendered as a Query/Output pair using the same query template as the live query, followed by the expected JSON output:

```
Query: {query text, as in S1.2}
Output: {"Likelihood": {region: value, ...}}
```

or, for few-shot-CoT:

```
Query: {query text, as in S1.2}
Output: {"Reasoning": "...", "Source": "...", "Likelihood": {region: value, ...}}
```

S2 Few-Shot Examples

Provenance note. SemioLLM's original evaluation used $k = 5$ clinician-authored few-shot examples that were not published alongside the paper. The $k = 3$ examples below are hand-written stand-ins created for this study's reproduction pipeline, matching the required schema, and are the exact examples used for every *fixed* few-shot / few-shot-CoT run reported in the

main text. They are not used by the retrieval-augmented conditions (few-shot-retrieved, RAOI-SC), which instead draw case-specific nearest neighbors from a held-out pool of the Semio2Brain dataset itself, as described in the main text.

S2.1 Example 1

```
Query: A patient comes into the clinic showing a semiological behaviour 'epigastric
↪  rising sensation followed by lip smacking and hand automatisms'. Determine the
↪  likelihood of a percentage of Seizure Onset Zones (SOZ) involved for the given
↪  list ['Temporal Lobe', 'Frontal Lobe', 'Cingulate Gyrus', 'Parietal Lobe',
↪  'Occipital Lobe', 'Insula', 'Hypothalamus'], such that postoperative seizure
↪  freedom is obtained.
Output: {"Reasoning": "Epigastric aura is a classic mesial temporal lobe symptom,
↪  often insular/opercular in origin, and the subsequent oroalimentary automatisms
↪  (lip smacking) and hand automatisms are hallmark features of mesial temporal
↪  lobe seizures.", "Source": "Beniczky et al. (2022). Seizure semiology: ILAE
↪  glossary of terms and their significance. Epileptic Disorders, 24(3), 447-495.",
↪  "Likelihood": {"Temporal Lobe": 80, "Frontal Lobe": 5, "Cingulate Gyrus": 0,
↪  "Parietal Lobe": 0, "Occipital Lobe": 0, "Insula": 15, "Hypothalamus": 0}}
```

S2.2 Example 2

```
Query: A patient comes into the clinic showing a semiological behaviour 'sudden
↪  bilateral arm extension with tonic posturing and preserved awareness'. Determine
↪  the likelihood of a percentage of Seizure Onset Zones (SOZ) involved for the
↪  given list ['Temporal Lobe', 'Frontal Lobe', 'Cingulate Gyrus', 'Parietal Lobe',
↪  'Occipital Lobe', 'Insula', 'Hypothalamus'], such that postoperative seizure
↪  freedom is obtained.
Output: {"Reasoning": "Bilateral tonic posturing with preserved awareness is
↪  characteristic of supplementary motor area seizures, a frontal lobe region with
↪  rapid bilateral spread through the SMA and cingulate motor circuits.", "Source":
↪  "Luders et al. (2006). The epileptogenic zone: general principles. Epileptic
↪  Disorders, 8:S1-S9.", "Likelihood": {"Temporal Lobe": 0, "Frontal Lobe": 85,
↪  "Cingulate Gyrus": 10, "Parietal Lobe": 0, "Occipital Lobe": 0, "Insula": 0,
↪  "Hypothalamus": 5}}
```

S2.3 Example 3

```
Query: A patient comes into the clinic showing a semiological behaviour 'brief
↪  flashing lights in the left visual field followed by eye blinking'. Determine
↪  the likelihood of a percentage of Seizure Onset Zones (SOZ) involved for the
↪  given list ['Temporal Lobe', 'Frontal Lobe', 'Cingulate Gyrus', 'Parietal Lobe',
↪  'Occipital Lobe', 'Insula', 'Hypothalamus'], such that postoperative seizure
↪  freedom is obtained.
Output: {"Reasoning": "Elementary visual hallucinations (flashing lights) localised
↪  to one hemifield are a specific sign of occipital lobe seizures, contralateral
↪  to the visual field affected.", "Source": "Beniczky et al. (2022). Seizure
↪  semiology: ILAE glossary of terms and their significance. Epileptic Disorders,
↪  24(3), 447-495.", "Likelihood": {"Temporal Lobe": 5, "Frontal Lobe": 0,
↪  "Cingulate Gyrus": 0, "Parietal Lobe": 5, "Occipital Lobe": 85, "Insula": 0,
↪  "Hypothalamus": 5}}
```

For plain few-shot (non-CoT) runs, each example above is rendered with only the `Likelihood` field in its `Output` (the `Reasoning` and `Source` fields are omitted from the worked example, matching the schema used for that strategy).

S3 Retrieval-Augmented and Permutation-Debiased Variants

Few-shot-retrieved and RAOI-SC use the identical query template, schema, and worked-example format described above (Sections S1.2–S1.4); the only differences are (i) which examples are selected – $k = 3$ nearest neighbors by TF-IDF cosine similarity to the query's semiology text, drawn from a held-out cross-validation pool, rather than the three fixed examples in Section S2 – and (ii) for the permutation-debiased strategies, the order of the seven candidate regions in `{regions}`, which is freshly and independently shuffled before each of the five self-consistency samples and applied consistently across that sample's query, schema, and (when combined with retrieval) worked-example output fields. Full implementation details are given in the main text, Section 3 (Materials and methods), and in the accompanying code repository.

S3.1 Consolidated Pseudocode

The following pseudocode makes explicit that RAOI-SC combines retrieval and candidate-order permutation within the same five-sample aggregation loop.

> **Inputs:** query text q; training pool D_{train}; canonical regions R; $k = 3$ retrieved examples; $m = 5$ samples.
> **Retrieve**(q, D_{train}, k): fit or use the TF-IDF representation defined on D_{train}; compute cosine similarity from q to every row; return the k highest-similarity labelled rows.
> **Predict**$(\mathit{condition}, q, D_{\text{train}})$: use retrieved examples when the condition requires retrieval, three fixed hand-written examples for fixed few-shot conditions, and no examples otherwise. Set the sample count to five for SC, SC-Perm, and RAOI-SC, and one otherwise. For each sample, shuffle R for SC-Perm and RAOI-SC, construct the prompt, generate at temperature 0.7 for five-sample conditions and 0 otherwise, and strictly parse the required region keys and numeric values. Reorder valid outputs canonically and return their componentwise median, renormalized to 100%. If no output is valid, record a fully-invalid row and apply the prespecified fallback.

S4 Reproducibility Metadata

All generation jobs ran on one NVIDIA H100 80-GB GPU per job. The server stack was vLLM 0.27.0, PyTorch 2.13.0+cu130, and Transformers 5.15.0, using automatic/bfloat16 dtype, tensor-parallel size 1, a maximum model context of 8,192 tokens, and a maximum completion length of 4,096 tokens. Client-side analysis used Python 3.9.25, httpx 0.28.1, OpenAI Python 2.48.0, NumPy 2.0.2, pandas 2.3.3, SciPy 1.13.1, and scikit-learn 1.6.1.

Exact model revisions are listed in Table S2.

Table S2: Exact Hugging Face model revisions evaluated.

**Model**	**Repository**	**Commit hash**
Qwen2.5-14B-Instruct	`Qwen/Qwen2.5-14B-Instruct`	`cf98f3b3bbb457ad9e2bb7baf9a0125b6b88caa8`
Mistral-7B-Instruct-v0.3	`mistralai/Mistral-7B-Instruct-v0.3`	`c170c708c41dac9275d15a8fff4eca08d52bab71`
Gemma-2-9B-it	`google/gemma-2-9b-it`	`11c9b309abf73637e4b6f9a3fa1e92e615547819`
Phi-4	`microsoft/phi-4`	`2db69c1c3e91a05d2c64a3185acfbaf36f744e25`
GLM-4-9B-Chat	`zai-org/glm-4-9b-chat-hf`	`8599336fc6c125203efb2360bfaf4c80eef1d1bf`
OpenBioLLM-8B	`aaditya/Llama3-OpenBioLLM-8B`	`70d6bb521cab6ca755b675ade38831eedf89d31c`
Med42-v2-8B	`m42-health/Llama3-Med42-8B`	`ceab7e7ee4b9dbde7ba82867f34274db51487d83`

Temperature was 0 for single-completion conditions and 0.7 for self-consistency conditions. No explicit top-p or model-side sampling seed was supplied; server defaults therefore applied. Fold creation, subsampling, and candidate permutations in the primary client run used seed

0. Prompts were assembled programmatically and were not manually truncated. No numerical citation-validity threshold was used. Folds, retrieval depth, sample count, temperature, and resampling settings were fixed for the final analysis; a systematic hyperparameter-sensitivity study was not performed.

S5 Paired Statistical Comparison and Bootstrap Procedure

This analysis compares SC and RAOI-SC on the same evaluation rows for each locally evaluated model. Correct-versus-incorrect predictions are compared with an exact two-sided McNemar test. We additionally report paired bootstrap differences for weighted-F1 and macro-F1. These paired analyses are descriptive comparisons of the completed evaluation campaign and do not assume independence between the two predictions for a given row.

All displayed 95% confidence intervals use standard bootstrap resampling of the full evaluation set: each of the 2,000 resamples contains N rows drawn with replacement from the original N rows. Confidence limits are the 2.5th and 97.5th percentiles of the resulting bootstrap distribution.

Table S3: Paired RAOI-SC minus SC results. Intervals are percentile 95% confidence intervals in percentage points; McNemar tests are exact two-sided tests on discordant top-1 correctness pairs.

Model	Δ weighted-F1	Δ macro-F1	Δ accuracy	McNemar p
Qwen2.5-14B	$+3.38\,[0.65, 6.09]$	$+0.09\,[-1.65, 1.68]$	-0.16	0.954
Mistral-7B	$+14.74\,[11.53, 17.82]$	$+8.66\,[6.08, 11.23]$	$+8.12$	4.27×10^{-7}
Gemma-2-9B	$+8.55\,[5.32, 11.81]$	$+7.65\,[5.04, 10.47]$	-3.15	0.0557
Phi-4	$+1.63\,[-1.20, 4.41]$	$-2.01\,[-5.06, 0.77]$	$+0.63$	0.711
GLM-4-9B	$+6.28\,[4.25, 8.15]$	$+4.01\,[2.37, 5.71]$	$+1.03$	0.223
OpenBioLLM-8B	$-3.58\,[-7.31, -0.05]$	$+2.60\,[0.51, 4.55]$	-18.14	4.90×10^{-24}
Med42-v2-8B	$+2.11\,[-1.15, 5.44]$	$+0.07\,[-4.25, 3.60]$	-8.75	6.04×10^{-8}

The number of folds, retrieved examples, self-consistency samples, temperature, and resampling iterations were fixed for the completed HPC experiment. A systematic sensitivity analysis over these hyperparameters was not performed and is therefore acknowledged as a limitation.

S6 Leakage and Retrieval Sensitivity Audit

In addition to exact self-retrieval checks, we audited repeated-text and publication-reference groups. The dataset contains 154 repeated-text groups covering 471 rows, including 66 groups with conflicting labels. The TF-IDF nearest-neighbour baseline achieved 55.72 weighted-F1 under the original row split, 50.43 after identical-text grouping, and 48.97 after publication-reference grouping. Patient identifiers were unavailable, so patient-level independence cannot be established.
